# Muscle atrophy in polytraumatized patients – a longitudinal observational pilot study

**DOI:** 10.3389/fphys.2025.1563380

**Published:** 2025-08-06

**Authors:** Danjana Teves, Torsten Pastor, Tatjana Pastor, Bergita Ganse

**Affiliations:** ^1^ Werner Siemens-Endowed Chair for Innovative Implant Development (Fracture Healing), Departments and Institutes of Surgery, Saarland University, Homburg, Germany; ^2^ Department of Orthopaedic and Trauma Surgery, Lucerne Cantonal Hospital, Lucerne, Switzerland; ^3^ Medical Faculty, University of Zurich (UZH), Zurich, Switzerland; ^4^ Department of Traumatology and Orthopaedics, Bürgerspital Solothurn, Solothurn, Switzerland; ^5^ Department of Trauma, Hand and Reconstructive Surgery, Departments and Institutes of Surgery, Saarland University, Homburg, Germany; ^6^ Center for Digital Neurotechnologies Saar (CDNS), Saarland University, Homburg, Germany

**Keywords:** bed rest, hand grip strength, immobilization, injury, intensive care, microcirculation, muscle perfusion, neuromuscular interaction

## Abstract

In polytraumatized patients, muscle atrophy appears more pronounced than in immobilized healthy study participants. However, rates and trajectories of the acute muscle atrophy and associated parameters have not been reported. In a prospective longitudinal pilot study with 10 patients (Injury Severity Score (ISS) ≥16), hand grip strength and inflammatory blood parameters were assessed. Skeletal muscle thickness of the rectus femoris (RF), vastus lateralis (VL), and tibialis anterior (TA), and subcutaneous tissue thickness over these muscles were measured via ultrasound. Muscle oxygen saturation (SO_2_), relative haemoglobin content (rHb), blood flow (BF), and blood flow velocity (BFV) were captured by laser-Doppler and white-light spectroscopy. Three women and seven men were included (age 43.2 ± 22.5 years; height 176.4 ± 5.7 cm; body weight 83.0 ± 14.5 kg; ISS 24.5 ± 4.6 points). Hand grip strength increased (p < 0.001) at a rate of 0.85%/d. Muscle thickness decreased (p < 0.001) at rates of −0.47%/d (RF), −0.39%/d (VL), and −0.38%/d (TA); no difference in the rate of decline between muscles (p = 0.908). Recovery of VL thickness was observed between the third and fourth week (p = 0.016). There were no changes in subcutaneous tissue thickness. Muscle perfusion parameters SO_2_, rHb, BF and BFV showed high variability with significant time effects only in the rHb of the TA (p = 0.003). CRP and leukocyte count decreased (both p < 0.001). Unexpectedly, grip strength increased despite a reduction in muscle thickness, likely after decreasing compared with pre-injury. Possible reasons are discussed.

## 1 Introduction

Polytraumatized patients are often bedridden for several weeks or even months due to their injuries. The sudden onset of immobilization, trauma and inflammation out of the patients’ regular daily life leads to muscle atrophy that is more pronounced in patients with trauma compared with immobilized healthy study participants of immobilization trials (Hardy et al., 2022). The additional inflammation caused by injury and illness leads to neuromuscular dysfunction by disturbing the neuromuscular interaction (Hermans and Van den Berghe, 2015). Trauma-related inflammation is characterized by an innate immune response as a reaction to shock, coagulopathy, hypothermia and soft tissue injury, that involves damage-associated and pathogen-associated molecular patterns (Pape et al., 2022). It includes proinflammatory mediators, i.e., tumour necrosis factor alpha, interleukin-1, and interleukin-6 (Hermans and Van den Berghe, 2015). More severely injured patients may experience a phenomenon called Intensive Care Unit Acquired Weakness (ICUAW), characterized by excessive muscle weakness and loss of physical function, including neuropathy and myopathy (Hermans and Van den Berghe, 2015; Chen and Huang, 2023). It is more frequent in patients with advanced age, female sex, and multiple organ failure (Fuentes-Aspe et al., 2024). This extensive muscle atrophy has been shown to be associated with poorer long-term results. For example, in severe trauma patients, the extent of the decrease in psoas area determined in computed tomography scans was strongly associated with poorer outcomes (Tazerout et al., 2022). In addition to these aspects, polytraumatized patients usually require medications such as pain killers and often sedation with additional effects on their overall performance, particularly in the initial period. Even though numerous clinical observational studies have been published that report the rates and trajectories of muscle atrophy in ICU patients in general (Hermans and Van den Berghe, 2015; Chen and Huang, 2023), to date no study has specifically dealt with polytraumatized patients. This patient group is usually younger and has less co-morbidities compared to other ICU patient groups. The authors were also interested in exploring, if polytraumatized patients could be used for their research instead of bed rest studies with healthy participants, that are a common analogue for spaceflight in space physiology and medicine (Pandiarajan and Hargens, 2020).

For polytraumatized patients, the rate and trajectory of the acute muscle atrophy and associated parameters have not yet been reported in a prospective longitudinal study. To obtain an overview of functional, morphometric, and physiological data, the following was hypothesized: 1. Declines in skeletal muscle strength and 2. Skeletal muscle thickness, 3. Increases in the thickness of the subcutaneous tissue layer, and 4. Due to the assumed relative increase in capillary density, increases in muscle oxygen saturation (SO_2_), relative haemoglobin content (rHb), blood flow (BF), and blood flow velocity (BFV).

## 2 Materials and methods

Ethical approval was obtained from the IRB of Saarland Medical Board (Ärztekammer des Saarlandes, application number 127/22). The study was registered in the German Clinical Trials Register (registration number DRKS00032012, registered 2 June 2023) and conducted according to the Declaration of Helsinki with written informed consent.

### 2.1 Patients

This longitudinal observational pilot study aimed to perform measurements in ten polytraumatized patients, as due to a lack of comparable human data in the literature, an *a priori* sample size calculation could not be conducted. Inclusion criteria were age 18 years and older, bone fracture with an Injury Severity Score (ISS) of 16 or more, written and verbal consent in the case of the capacity to consent, or otherwise consent by a legal guardian. The exclusion criteria were age younger than 18 years, an ISS under 16, or a lack of verbal and written informed consent. The ISS was assessed for each patient based on the Abbreviated Injury Scale (AIS) for the three most severely injured body regions (Baker et al., 1974; Greenspan et al., 1985).

### 2.2 Measurements

All measurements were conducted by the first author between April and July of 2023. Measurements were repeated every two to three days, if the current patient status allowed them, and if the measurements did not interfere with the clinical treatment. Hand grip strength was measured with a hand dynamometer (Kern MAP 130K1, Kern, Balingen, Germany). The patients, if awake, were asked to conduct three maximal voluntary contractions in supine position with a rest period in-between measurements and with the elbow bent at 90°. The highest of the three values was used for statistical analysis.

Muscle thickness and the thickness of the subcutaneous fat layer of the rectus femoris (RF), tibialis anterior (TA), and vastus lateralis (VL) muscles were measured by ultrasound (Mindray MX7, Shenzhen, China) with an L13-3Ns ultrasonic transducer (frequency 3.0 MHz–13.0 MHz) and analysed using ImageJ (National Institutes of Health, Bethesta, Maryland, United States, https://imagej.net/ij/). The ultrasound measurements of the RF were taken at 50% of a straight line between spina iliaca anterior superior and the centre of the knee cap, and the VL measurement was taken 10 cm lateral of that point. For the TA, measurements were performed between the middle and proximal third of a line between the bottom tip of the knee cap and the lateral malleolus.

Muscle perfusion was assessed with the device ‘Oxygen to see’ (O2C, LEA Medizintechnik, Giessen, Germany) in 14 mm depth. The device uses laser-Doppler flowmetry and white light spectroscopy to deliver the parameters SO_2_, rHb, BF, and BFV. Measurements were taken for the RF and TA in the same locations as the ultrasound measurements. Details on the device and its measurement principle can be found in a recent paper (Scholz et al., 2025). Three measurements were taken each, the second and third 2 cm proximal and distal of the first. The three values of each parameter were averaged for analysis.

The inflammatory blood markers C-reactive protein (CRP) and the leukocyte count were determined as clinical routine parameters and these values were used in this study.

### 2.3 Statistical analyses

All the statistical tests were conducted in IBM SPSS Statistics version 29 (IBM SPSS Statistics, Armonk, NY, United States). Significance was defined as p < 0.05. Linear mixed effect (LME) models were fitted to analyse time effects with time as a fixed effect and patient as a random effect; a Bonferroni adjustment was made for multiple comparisons. The LME analyses were conducted based on weekly time periods. Missing data were excluded from the analyses. R^2^ values as a measure of variance and relative change rates (in %/d) were determined by linear regression. In addition, for muscle thickness, a univariate ANOVA was used to determine differences in the decline rates between muscles. Previously, normal distribution was confirmed with the Kolmogorov-Smirnov test.

## 3 Results

**FIGURE 1:**
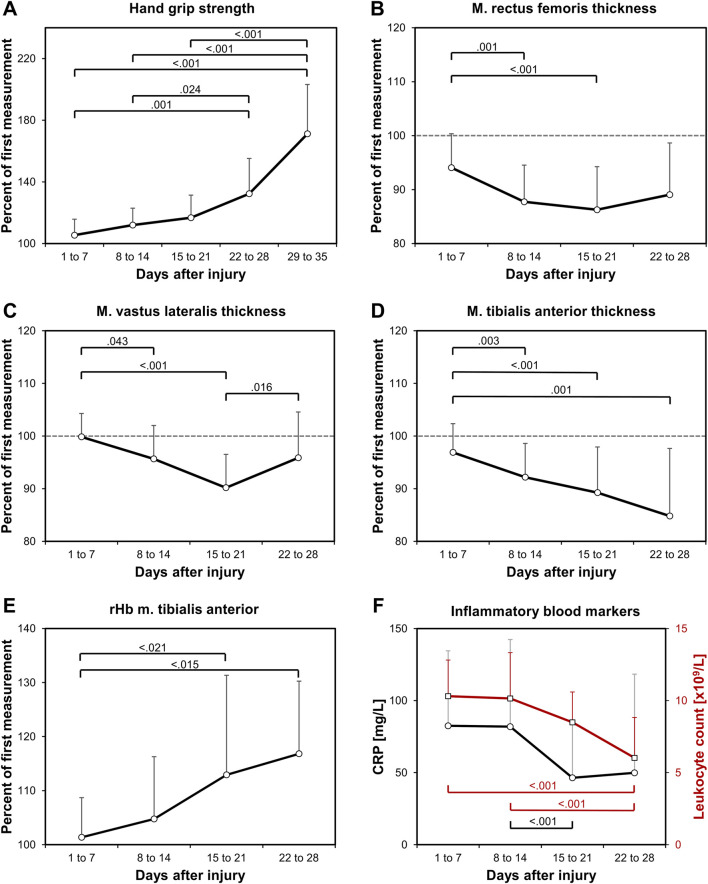
Study findings. Relative longitudinal changes in **(A)** hand grip strength, and in the muscle thickness of **(B)** the RF, **(C)** the VL, **(D)** the TA, as well as in **(E)** the rHb of the TA, all normalized to the first value of each patient. In addition, **(F)** blood CRP and leukocyte count values are shown in absolute values to indicate the decrease in trauma-related systemic inflammation. Significant p values are shown from the LME-models pair-wise analyses.

Three women and seven men were included (age 43.2 ± 22.5 years; height 176.4 ± 5.7 cm; body weight 83.0 ± 14.5 kg; Injury Severity Score 24.5 ± 4.6 points). Table 1 shows R^2^ and p values for all outcome parameters of each muscle. All significant findings of the pair-wise analyses with p values are indicated in Figure 1.

**TABLE 1:** Variability and p values for each outcome parameter and each muscle.

	RF		VL		TA	
Parameter	R^2^ value	P Value	R^2^ value	P Value	R^2^ value	P Value
Muscle thickness	0.39	**<0.001**	0.36	**<0.001**	0.39	**<0.001**
Subcutaneous tissue thickness	0.38	0.819	0.41	0.081	0.23	0.127
SO_2_	0.05	0.593	n/a	n/a	0.21	0.105
rHb	0.18	0.805	n/a	n/a	0.31	**0.003**
BF	0.29	0.240	n/a	n/a	0.26	0.119
BFV	0.35	0.153	n/a	n/a	0.31	0.487

Outcome parameters for the rectus femoris (RF), vastus lateralis (VL, if available) and tibialis anterior (TA) muscles. R^2^ is shown as a measure of variability. P values of the LME, model univariate tests are shown and are highlighted in bold, if significant. The ‘n/a’ indicates that the data were not collected.

Hand grip strength increased significantly (p < 0.001; R^2^ = 0.43) at a growing rate (Figure 1A). On average, the increase in hand grip strength comprised 0.85%/d. Muscle thickness decreased significantly in all muscles at rates of −0.47%/d (RF), −0.39%/d (VL), and −0.38%/d (TA) (Figures 1B–D). The decline showed no significant differences in the rate of decline between the muscle groups (p = 0.908). After the initial decline, a significant increase in muscle thickness between the third and fourth week was observed for the VL (p = 0.016, Figure 1C), but not for the RF or the TA, which indicates an earlier recovery of the VL. There were no changes in the subcutaneous tissue thickness. The analysed muscle perfusion parameters SO_2_, rHb, BF and BFV showed a high variability with significant time effects only in the rHb of the TA (p = 0.003, Figure 1E). There were no significant changes in the RF perfusion data. The CRP blood value (p = 0.025) and the leukocyte count (p < 0.001) significantly decreased (Figure 1F).

## 4 Discussion

This pilot study showed increasing hand grip strength, decreasing muscle thickness and unchanged subcutaneous tissue thickness. Muscle perfusion parameters had a high variability. Only an increase in rHb of the TA was significant. Blood inflammatory markers decreased.

Immobilization-induced skeletal muscle atrophy is characterized by decreases in myofiber cross-sectional area and a transition from slow to fast fibre types with increased hybrid fibre-type muscle cells, called a fibre-type shift (Blottner et al., 2014; Caiozzo et al., 1994; Widrick et al., 1999). The extent of the decline in fibre cross-sectional area depends on the fibre type, and its composition differs among skeletal muscles (Edgerton et al., 1995). Based on differences in fibre type composition, atrophy rates were highest in the triceps surae muscles (−11.2% after 28 days of immobilisation), followed by quadriceps (−9.2%), hamstrings (−6.5%), and foot dorsiflexors (−3.2%) (Hardy et al., 2022). The present results are in line with this; however, the difference in the rate of decline between these muscles was not significant, likely due to the low patient number. Muscle thickness decreased the fastest in the RF (−0.47%/d), followed by the VL (−0.39%/d), which are both quadriceps muscles, and less in the TA (−0.38%/d), a foot dorsiflexor. Throughout immobilisation, the rate of atrophy usually decreases (Hardy et al., 2022; Marusic et al., 2021). However, in this study, increasing activity led to VL recovery while a further decline was observed in the TA. Due to the low patient number, sex differences in the rate of decline in muscle thickness could not be addressed in this study. Nevertheless, this was not the aim of this study anyway. It is known that women in the ICU experience atrophy rates approximately three times higher than men (Wu et al., 2022). The methodology of using ultrasound measurements to quantify muscle thickness has certainly proven feasible in this study. Further methods exist and it is a matter of a debate in the current literature, which method is most appropriate for use in intensive care patients (Hernández-Socorro et al., 2021).

Increasing hand grip strength was an unexpected finding, as muscle atrophy is commonly associated with decreasing muscle strength. Losses in muscle strength usually exceed losses in muscle volume (Marusic et al., 2021; Wu et al., 2022; Hernández-Socorro et al., 2021; Kramer et al., 2021; Pišot et al., 2016). This finding may be explained by initial sedation, pain and pain medication, potential learning effects in repeated grip strength testing, and delayed neuromuscular recovery despite muscle wasting, as well as possibly by a weakness caused by impaired neuromuscular interaction due to inflammation (Hermans and Van den Berghe, 2015). Neuromuscular junction impairment and mitochondrial dysfunction are known causes of sarcopenia in ageing (Miao et al., 2024), however, in the present cohort, this impairment was only temporary. These acute effects likely led to a much lower initial muscle strength than before the injury, and the increase in muscle strength that was observed in this study would then reflect the recovery from these influences. This rebound has a greater effect than the immobilization-related reduction in strength, therefore resulting in a net increase.

The decrease in the cross-sectional area of muscle cells is usually associated with a relative increase in capillary density (Bosutti et al., 2015; Hendrickse et al., 2022). In addition, an intracellular nutrient overload rapidly leads to skeletal muscle insulin insensitivity and mitochondrial alterations, as well as to accumulation of lipotoxic ceramides and sphingomyelins (Eggelbusch et al., 2024). For these reasons, and as injury and surgery are associated with initial blood loss and effects of medications and inflammation, the authors expected increases in subcutaneous tissue thickness and muscle perfusion. This was, however, not the case. The increase found in the rHb of the TA muscle may be either due to the suspected increase in capillary density or due to an overall increase in Hb due to haematopoiesis following blood loss. An increase in capillary density with muscle atrophy could have led to increased muscle oxygenation and blood flow, but this was not the case either. The variation among patients and time points was very high and muscle perfusion measurements will likely only reveal greater effects in a large patient collective. Among the possible reasons for the high variation are effects of different treatments and injury patterns, varying fluid administration and intake, circulatory status, individual metabolic differences, and inflammatory responses to the injuries (Pape et al., 2022). The trajectories of the decreases in CRP and the leukocyte count are in line with the literature and reflect the typical trajectories after injury when no additional bacterial infection appears (Niggli et al., 2025). Indeed, the initial increase in CRP and the leucocyte count is rather rapid and followed by a maximum and a slower decrease.

With regard to the question whether physiological studies in polytraumatized patients could at least for some research questions replace bed rest studies with healthy participants, the answer is that the great variability and the unexpected increase in hand grip strength would likely make this difficult for most research questions. A large number of patients would be required. This indicates and underlines the importance of bed rest studies conducted with healthy participants for physiological research.

The main limitation of this pilot study is its small sample size. Therefore, the findings must be interpreted with caution. Another limitation is that the measurements were not conducted on the same days in all patients, which is an operational challenge and should be considered in future studies. In addition, a limitation of this study is that inter-rater reliability testing has not been performed for the ultrasound and perfusion measurements. In this study, all measurements were performed by the same person, but inter-rater reliability is certainly an important aspect that needs to be considered in future studies.

In conclusion, hand grip strength increased and muscle thickness decreased in this pilot trial of polytraumatized patients. Studies with more patients may lead to more clarity regarding changes in subcutaneous fat layer thickness and muscle perfusion parameters.

## Data Availability

The datasets presented in this article are not readily available because the requesting institution needs to fall within the eligibility criteria of German data protection law. The reason is that these are patient data and the law forces us to applying this procedure. Requests to access the datasets should be directed to the corresponding author.

## References

[B1] Baker S. P. O'Neill B. Haddon W. Jr Long W. B. (1974). The injury severity score: a method for describing patients with multiple injuries and evaluating emergency care. J. Trauma 14 (3), 187–196. 4814394

[B2] Blottner D. Bosutti A. Degens H. Schiffl G. Gutsmann M. Buehlmeier J. (2014). Whey protein plus bicarbonate supplement has little effects on structural atrophy and proteolysis marker immunopatterns in skeletal muscle disuse during 21 days of bed rest. J. Musculoskelet. Neuronal Interact. 14 (4), 432–444. 25524969

[B3] Bosutti A. Egginton S. Barnouin Y. Ganse B. Rittweger J. Degens H. (2015). Local capillary supply in muscle is not determined by local oxidative capacity. J. Exp. Biol. 218 (Pt 21), 3377–3380. 10.1242/jeb.126664 26385326

[B4] Caiozzo V. J. Baker M. J. Herrick R. E. Tao M. Baldwin K. M. (1994). Effect of spaceflight on skeletal muscle: mechanical properties and myosin isoform content of a slow muscle. J. Appl. Physiol. 76 (4), 1764–1773. 10.1152/jappl.1994.76.4.1764 8045858

[B5] Chen J. Huang M. (2023). Intensive care unit-acquired weakness: recent insights. J. Intensive Med. 4 (1), 73–80. 10.1016/j.jointm.2023.07.002 38263973 PMC10800771

[B6] Edgerton V. R. Zhou M. Y. Ohira Y. Klitgaard H. Jiang B. Bell G. (1995). Human fiber size and enzymatic properties after 5 and 11 days of spaceflight. J. Appl. Physiol. 78 (5), 1733–1739. 10.1152/jappl.1995.78.5.1733 7649906

[B7] Eggelbusch M. Charlton B. T. Bosutti A. Ganse B. Giakoumaki I. Grootemaat A. E. (2024). The impact of bed rest on human skeletal muscle metabolism. Cell Rep. Med. 5 (1), 101372. 10.1016/j.xcrm.2023.101372 38232697 PMC10829795

[B8] Fuentes-Aspe R. Gutierrez-Arias R. González-Seguel F. Marzuca-Nassr G. N. Torres-Castro R. Najum-Flores J. (2024). Which factors are associated with acquired weakness in the ICU? An overview of systematic reviews and meta-analyses. J. Intensive Care 12 (1), 33. 10.1186/s40560-024-00744-0 39232808 PMC11375885

[B9] Greenspan L. McLellan B. A. Greig H. (1985). Abbreviated injury scale and injury severity score: a scoring chart. J. Trauma 25 (1), 60–64. 10.1097/00005373-198501000-00010 3965737

[B10] Hardy E. J. O. Inns T. B. Hatt J. Doleman B. Bass J. J. Atherton P. J. (2022). The time course of disuse muscle atrophy of the lower limb in health and disease. J. Cachexia Sarcopenia Muscle 13 (6), 2616–2629. 10.1002/jcsm.13067 36104842 PMC9745468

[B11] Hendrickse P. W. Wust R. Ganse B. Rittweger J. Giakoumaki I. Bosutti A. (2022). Capillary rarefaction during bed rest is proportionally less than fibre atrophy and loss of oxidative capacity. J. Cachexia Sarcopenia Muscle 13 (6), 2712–2723. 10.1002/jcsm.13072 36102002 PMC9745458

[B12] Hermans G. Van den Berghe G. (2015). Clinical review: intensive care unit acquired weakness. Crit. Care 19 (1), 274. 10.1186/s13054-015-0993-7 26242743 PMC4526175

[B13] Hernández-Socorro C. R. Saavedra P. López-Fernández J. C. Lübbe-Vazquez F. Ruiz-Santana S. (2021). Novel high-quality sonographic methods to diagnose muscle wasting in long-stay critically ill patients: shear wave elastography, superb microvascular imaging and contrast-enhanced ultrasound. Nutrients 13 (7), 2224. 10.3390/nu13072224 34209526 PMC8308272

[B14] Kramer A. Venegas-Carro M. Zange J. Sies W. Maffiuletti N. A. Gruber M. (2021). Daily 30-min exposure to artificial gravity during 60 days of bed rest does not maintain aerobic exercise capacity but mitigates some deteriorations of muscle function: results from the AGBRESA RCT. Eur. J. Appl. Physiol. 121 (7), 2015–2026. 10.1007/s00421-021-04673-w 33811556 PMC8192329

[B15] Marusic U. Narici M. Simunic B. Pisot R. Ritzmann R. (2021). Nonuniform loss of muscle strength and atrophy during bed rest: a systematic review. J. Appl. Physiol. 131 (1), 194–206. 10.1152/japplphysiol.00363.2020 33703945 PMC8325614

[B16] Miao Y. Xie L. Song J. Cai X. Yang J. Ma X. (2024). Unraveling the causes of sarcopenia: roles of neuromuscular junction impairment and mitochondrial dysfunction. Physiol. Rep. 12 (1), e15917. 10.14814/phy2.15917 38225199 PMC10789655

[B17] Niggli C. Vetter P. Hambrecht J. Pape H. C. Mica L. (2025). Sex differences in the time trends of sepsis biomarkers following polytrauma. Sci. Rep. 15 (1), 2398. 10.1038/s41598-025-86495-w 39827304 PMC11742873

[B18] Pandiarajan M. Hargens A. R. (2020). Ground-based analogs for human spaceflight. Front. Physiol. 11, 716. 10.3389/fphys.2020.00716 32655420 PMC7324748

[B19] Pape H. C. Moore E. E. McKinley T. Sauaia A. (2022). Pathophysiology in patients with polytrauma. Injury 53 (7), 2400–2412. 10.1016/j.injury.2022.04.009 35577600

[B20] Pišot R. Marusic U. Biolo G. Mazzucco S. Lazzer S. Grassi B. (2016). Greater loss in muscle mass and function but smaller metabolic alterations in older compared with younger men following 2 wk of bed rest and recovery. J. Appl. Physiol. 120 (8), 922–929. 10.1152/japplphysiol.00858.2015 26823343

[B21] Scholz O. Nowicki C. Warmerdam E. Rother S. Ganse B. (2025). New sensor options for smart fracture implants and wearable devices: laser-doppler and white-light spectroscopy allow monitoring of bone regeneration *via* perfusion measurement. Biosens. Bioelectron. 280, 117442. 10.1016/j.bios.2025.117442 40199098

[B22] Tazerout S. Martinez O. Monsonis B. Millet I. Taourel P. Capdevila X. (2022). Acute post-traumatic muscle atrophy on CT scan predicts prolonged mechanical ventilation and a worse outcome in severe trauma patients. Injury 53 (7), 2501–2510. 10.1016/j.injury.2022.05.005 35613963

[B23] Widrick J. J. Knuth S. T. Norenberg K. M. Romatowski J. G. Bain J. L. Riley D. A. (1999). Effect of a 17 day spaceflight on contractile properties of human soleus muscle fibres. J. Physiol. 516, 915–930. 10.1111/j.1469-7793.1999.0915u.x 10200437 PMC2269300

[B24] Wu R. Y. Sung W. H. Cheng H. C. Yeh H. J. (2022). Investigating the rate of skeletal muscle atrophy in men and women in the intensive care unit: a prospective observational study. Sci. Rep. 12 (1), 16629. 10.1038/s41598-022-21052-3 36198744 PMC9534861

